# Prevalence and associated factors of vitamin B12 deficiency among Vietnamese patients with type 2 diabetes receiving metformin therapy: a cross-sectional study

**DOI:** 10.3389/fcdhc.2026.1924740

**Published:** 2026-10-16

**Authors:** Thi Kim Dung Nguyen, Thi Lan Anh Ke, Van Linh Pham, Xuan Thuy Tran, Thanh Binh Vu

**Affiliations:** 1 Department of Internal Medicine, Thai Binh University of Medicine and Pharmacy, Hung Yen, Vietnam; 2 Department of Internal Medicine, Hai Phong University of Medicine and Pharmacy, Hai Phong, Vietnam; 3 Department of Pathophysiology, Hai Phong University of Medicine and Pharmacy, Hai Phong, Vietnam

**Keywords:** anemia, cross-sectional study, metformin, type 2 diabetes mellitus, Vietnam, vitamin B12 deficiency

## Abstract

**Background/objectives:**

This study examined serum vitamin B12 status and associated clinical factors among Vietnamese patients with type 2 diabetes mellitus (T2DM) receiving metformin.

**Methods:**

This hospital-based cross-sectional study included 400 eligible patients treated with metformin for at least 6 months and recruited by non-probability convenience sampling at two hospitals in Thai Binh, Vietnam. The study-defined outcome was serum vitamin B12 <300 pg/mL, comprising absolute deficiency (<200 pg/mL) and marginal status (200 to <300 pg/mL). Associations were examined using univariable analysis and the reported exploratory multivariable logistic regression model.

**Results:**

Overall, 103 participants (25.75%) met the combined threshold: 29 (7.25%) had absolute deficiency and 74 (18.50%) had marginal status. In the reported adjusted model, metformin use >5 years (adjusted odds ratio [AOR] = 1.84; 95% confidence interval [CI]: 1.07-3.18) and anemia (AOR = 3.43; 95% CI: 1.62-7.23) were associated with the outcome. The macrocytosis estimate was highly imprecise.

**Conclusions:**

Low serum vitamin B12 was common in this hospital-based sample. Periodic assessment merits consideration during prolonged metformin therapy, with use >5 years serving as a pragmatic prompt rather than a validated testing threshold. Unexplained anemia or neuropathic symptoms warrant assessment when deficiency is suspected, regardless of treatment duration. The adjusted findings remain exploratory and do not establish causality or model robustness.

## 1 Introduction

Metformin remains an important treatment for type 2 diabetes mellitus (T2DM), within an individualized approach that considers comorbidities and treatment goals (1). Its association with reduced vitamin B12 status is supported by evidence beyond cross-sectional surveys. In a randomized placebo-controlled trial of insulin-treated patients with T2DM, de Jager et al. observed a greater occurrence of biochemical vitamin B12 deficiency during long-term metformin treatment (2). The Diabetes Prevention Program Outcomes Study also associated longer cumulative metformin exposure with low vitamin B12 status; however, that population was originally recruited for diabetes prevention rather than treatment of established T2DM (3). These complementary designs support attention to vitamin B12 during prolonged treatment while requiring population-specific interpretation.

Impairment of calcium-dependent absorption of the vitamin B12-intrinsic factor complex is one proposed mechanism. Bauman et al. demonstrated reversal of metformin-associated malabsorption after calcium supplementation in a small physiological study (4). This provides mechanistic support, rather than evidence for routine calcium supplementation. Clinical relevance extends beyond anemia because symptoms of vitamin B12 deficiency may overlap with diabetic neuropathy. Ahmed et al. did not identify a significant association between biochemical deficiency and peripheral neuropathy in their cross-sectional study (5), whereas Didangelos et al. reported improvement in several neurological measures after vitamin B12 supplementation in a randomized trial of selected metformin-treated patients with neuropathy (6). Thus, a low serum result and clinically manifest deficiency should not be treated as interchangeable outcomes.

Comparisons of prevalence require attention to serum thresholds, supplementation, and sampling. Kim et al. reported vitamin B12 deficiency in 22.2% of 1, 111 metformin-treated patients in Korea using a threshold of <300 pg/mL (7). In a multicenter Chinese study, Gao et al. evaluated definite and borderline categories separately and found associations with higher daily metformin doses, but not with their selected duration categories (8). Such differences in exposure definitions and case classification can affect both the observed frequency of low vitamin B12 and the factors associated with it.

Vietnamese evidence is also heterogeneous. Huynh et al. reported a prevalence of 18.6% in 156 patients, with metformin dose retaining an association in adjusted analyses (9). Phan et al. reported 22.1% among 145 patients at a tertiary hospital in southern Vietnam and identified adjusted associations with treatment duration, dose, and HbA1c (10). At Friendship Hospital, Vu et al. studied elderly patients in a setting where vitamin B12 supplementation was common, limiting direct comparison with samples excluding supplement users (11). Further evidence from northern Vietnam can therefore extend, rather than replace, these studies and clarify the clinical correlates observed under different eligibility criteria.

The American Diabetes Association and the UK Medicines and Healthcare products Regulatory Agency advise consideration of periodic vitamin B12 assessment during long-term metformin treatment, particularly in patients with risk factors or suggestive hematological or neurological findings (12, 13). The present study aimed to estimate the proportion of patients meeting a study-defined serum vitamin B12 threshold of <300 pg/mL at two hospitals in Thai Binh, describe absolute and marginal categories separately, and examine demographic, treatment-related, lifestyle, and hematological associations. The intention was to identify clinical correlates in this setting, not to establish causation, demonstrate a sex-specific mechanism, or validate a screening algorithm.

## 2 Materials and methods

### 2.1 Study design and setting

A cross-sectional observational study was conducted at the Department of Internal Medicine, Thai Binh Medical University Hospital, and the Department of Endocrinology, Thai Binh General Hospital. The study period was from January 2024 to September 2025. The reporting of this study was guided by the Strengthening the Reporting of Observational Studies in Epidemiology (STROBE) statement (14).

### 2.2 Study participants

The study enrolled 400 patients diagnosed with T2DM who met the following criteria.

#### 2.2.1 Inclusion criteria

Patients diagnosed with T2DM according to the Vietnamese Ministry of Health guidelines (2020) (15), currently treated with metformin for at least 6 months, and consented to participate in the study.

#### 2.2.2 Exclusion criteria

Patients with conditions affecting vitamin B12 absorption, including gastrectomy, ileal resection, Crohn disease, or celiac disease; patients using medications affecting vitamin B12 absorption for more than 12 months, including proton pump inhibitors, H2 receptor antagonists, or colchicine; patients with pernicious anemia or malignancies; strict vegetarians; and patients currently taking vitamin B12 supplements.

### 2.3 Sample size and sampling method

The minimum sample size for estimating a single proportion was calculated as n = Z²p(1 - p)/d², using an anticipated prevalence of 22.2% based on Kim et al. (7), a 95% confidence level (Z = 1.96), and an absolute precision of 5% (d = 0.05). The calculation yielded 265.40, corresponding to a minimum of 266 participants after rounding upward. The final sample comprised 400 eligible patients.

Non-probability convenience sampling was used at the two participating hospitals. Patients were included if they met the stated eligibility criteria and provided informed consent. This was not a random or population-based sample.

### 2.4 Measurements and data collection

Data were collected through direct interviews and medical record reviews using a structured questionnaire, followed by clinical examination and laboratory testing.

Demographic and lifestyle factors included age (categorized as ≤60 and >60 years), sex (male/female), smoking status (yes/no), alcohol consumption (yes/no), and physical exercise (yes/no). Alcohol consumption was self-reported and analyzed as a binary variable; this measure did not characterize quantity, duration, or drinking pattern.

Clinical characteristics included duration of diabetes (<5, 5-10, and >10 years) and presence of other chronic diseases, such as hypertension and dyslipidemia.

Metformin use was assessed according to duration of use (≤5 years and >5 years), daily dose (<1000, 1000-1500, and >1500 mg/day), and use in combination with other anti-diabetic drugs, such as sulfonylureas or insulin.

Vitamin B12 status was determined using serum vitamin B12 concentration. The study-defined combined outcome was <300 pg/mL, comprising absolute deficiency (<200 pg/mL) and marginal status (200 to <300 pg/mL); values ≥300 pg/mL were outside the combined category. This serum-based definition includes marginal values and does not, by itself, establish functional deficiency.

Anemia was defined as hemoglobin <130 g/L in men and <120 g/L in women, consistent with the conventional WHO thresholds for adult men and non-pregnant women (16). Macrocytosis was defined separately as a mean corpuscular volume (MCV) >100 fL.

Glycemic control was classified according to glycated hemoglobin (HbA1c) level, with HbA1c <7.0% considered controlled and HbA1c ≥7.0% considered uncontrolled.

### 2.5 Statistical analysis

The original analysis used EpiData for data entry and SPSS version 27.0 (IBM Corp., Armonk, NY, USA) for statistical analysis. Categorical variables were summarized as frequencies and percentages. During revision, the group comparisons in **Tables 1**, **2** were recomputed from the displayed frequencies using Pearson chi-square tests without continuity correction, or two-sided Fisher exact tests for 2 x 2 tables with an expected cell count <5. The corresponding crude odds ratios in **Table 3** were recalculated by the cross-product method with log-scale Wald 95% confidence intervals and two-sided Wald p-values.

**Table 1:** General characteristics of the study population according to vitamin B12 deficiency status.

Characteristic	Category	B12 ≥300 n	B12 ≥300%	B12 <300 n	B12 <300%	P-value
Age group	>60	239	73.1	88	26.9	0.261
Age group	≤60	58	79.5	15	20.5	
Sex	Male	135	69.2	60	30.8	0.025
Sex	Female	162	79.0	43	21.0	
Duration of diabetes	<5 years	71	83.5	14	16.5	0.038
Duration of diabetes	5–10 years	127	74.7	43	25.3	
Duration of diabetes	>10 years	99	68.3	46	31.7	
Other chronic diseases	No	8	80.0	2	20.0	1.000*
Other chronic diseases	Yes	289	74.1	101	25.9	
Smoking	No	249	75.9	79	24.1	0.104
Smoking	Yes	48	66.7	24	33.3	
Alcohol consumption	No	245	77.3	72	22.7	0.007
Alcohol consumption	Yes	52	62.7	31	37.3	
Physical exercise	No	37	84.1	7	15.9	0.114
Physical exercise	Yes	260	73.0	96	27.0	

The outcome is the study-defined combined category, serum vitamin B12 <300 pg/mL. p-values were recomputed from the displayed counts using Pearson chi-square tests without continuity correction; * denotes a two-sided Fisher exact test because an expected cell count was <5. n, number of patients.

**Table 2:** Characteristics of drug use according to vitamin B12 deficiency status.

Characteristic	Category	B12 ≥300 n	B12 ≥300%	B12 <300 n	B12 <300%	P-value
Duration of metformin use	≤5 years	98	81.7	22	18.3	0.026
Duration of metformin use	>5 years	199	71.1	81	28.9	
Daily metformin dose	<1000 mg/day	44	75.9	14	24.1	0.297
Daily metformin dose	1000–1500 mg/day	56	81.2	13	18.8	
Daily metformin dose	>1500 mg/day	197	72.2	76	27.8	
Combination therapy	No	36	72.0	14	28.0	0.697
Combination therapy	Yes	261	74.6	89	25.4	
Concomitant medications	No	9	69.2	4	30.8	0.748*
Concomitant medications	Yes	288	74.4	99	25.6	

The outcome is the study-defined combined category, serum vitamin B12 <300 pg/mL. p-values were recomputed from the displayed counts using Pearson chi-square tests without continuity correction; * denotes a two-sided Fisher exact test because an expected cell count was <5. n, number of patients.

**Table 3:** Univariable analysis of factors associated with vitamin B12 deficiency.

Variable	Category/reference	Univariable OR (95% CI)	P-value
Age group	≤60	1.0	
	>60	1.42 (0.77-2.64)	0.263
Sex	Female	1.0	
	Male	1.67 (1.06-2.63)	0.026
Duration of metformin use	≤5 years	1.0	
	>5 years	1.81 (1.07-3.08)	0.028
Daily metformin dose	<1000 mg/day	1.0	
	1000–1500 mg/day	0.73 (0.31-1.71)	0.468
Daily metformin dose	<1000 mg/day	1.0	
	>1500 mg/day	1.21 (0.63-2.34)	0.566
HbA1c control	≥7.0%	1.0	
	<7.0%	0.8 (0.5-1.3) †	0.379
Anemia	No	1.0	
	Yes	2.5 (1.3-5.1) †	0.008
Macrocytosis	No	1.0	
	Yes	8.9 (1.0-86.3) †	0.055
Smoking	No	1.0	
	Yes	1.58 (0.91-2.74)	0.106
Alcohol consumption	No	1.0	
	Yes	2.03 (1.21-3.40)	0.007
Physical exercise	Yes	1.0	
	No	0.51 (0.22-1.19)	0.119

CI, confidence interval; HbA1c, glycated hemoglobin; OR, odds ratio. The outcome is serum vitamin B12 <300 pg/mL. Estimates without a dagger were recalculated from the displayed counts using cross-product odds ratios and log-scale Wald confidence intervals and p-values. † Original reported estimates for HbA1c, anemia, and macrocytosis are retained because their underlying cell counts were not available for count-based verification. Reference groups are explicitly stated; no patient-level reclassification was performed.

The reported exploratory multivariable model comprised metformin use >5 years, anemia, male sex, alcohol consumption, and macrocytosis. The first four variables met the nominal p < 0.05 threshold in the reported univariable analyses; macrocytosis did not (p = 0.055). For transparent reporting of the original fitted model, the macrocytosis coefficient is retained in **Table 4** and interpreted as an exploratory hematological correlate. This reporting rationale does not establish that its inclusion was prespecified. Daily metformin dose and diabetes duration were not included in the reported adjusted model; therefore, the adjusted associations cannot be interpreted as independent of these characteristics.

**Table 4:** Originally reported exploratory multivariable model of vitamin B12 status.

Related factor	AOR	95% CI	P-value
Metformin use >5 years	1.84	1.07-3.18	0.028
Anemia	3.43	1.62-7.23	0.001
Male sex	1.62	0.93-2.83	0.090
Alcohol consumption	1.31	0.94-1.83	0.110
Macrocytosis (exploratory)	7.91	0.79-79.37	0.078

AOR, adjusted odds ratio; CI, confidence interval. The model includes all five factors shown. Contrasts are >5 versus ≤5 years of metformin use, anemia versus no anemia, male versus female, alcohol consumption versus none, and macrocytosis versus no macrocytosis. All coefficients and intervals are retained from the source manuscript, not re-estimated during this revision. The macrocytosis estimate is highly imprecise; stability of the other AORs after excluding this variable has not been established.

The recalculations used tabulated counts, not a reconstructed patient-level dataset. The HbA1c, anemia, and macrocytosis crude estimates and the multivariable coefficients were retained as originally reported and were not independently re-estimated during this revision. No refitted model, bootstrap validation, or analysis excluding macrocytosis is presented. Confidence-interval width is used to describe precision, not as proof of model stability. All p-values are two-sided, with a nominal significance threshold of 0.05. Odds ratios represent associations rather than causal risk ratios.

### 2.6 Ethical considerations

The study was conducted in accordance with the ethical principles of the Declaration of Helsinki (17). Ethical approval was granted by the Ethics Council of Thai Binh University of Medicine and Pharmacy (Decision No. 0424, dated 21 April 2024). All participants provided written informed consent, and their personal information was kept confidential.

## 3 Results

The prevalence of vitamin B12 deficiency differed significantly according to sex, duration of diabetes, and alcohol consumption (p < 0.05). Specifically, the rate was higher in men (30.8%), in patients reporting alcohol consumption (37.3%), and tended to increase with diabetes duration, from 16.5% in the group with diabetes duration <5 years to 31.7% in the group with diabetes duration >10 years. Age group, smoking, physical exercise, and comorbidity status were not significantly associated with vitamin B12 deficiency (p > 0.05) (**Table 1**).

Duration of metformin use was significantly associated with vitamin B12 deficiency (p < 0.05), with a higher deficiency rate among patients using metformin for more than 5 years (28.9%) compared with those using it for 5 years or less (18.3%). No statistically significant differences were found in the rate of vitamin B12 deficiency according to daily metformin dose, combination therapy, or concomitant medication use (p > 0.05) (**Table 2**).

Of the 400 participants, 103 (25.75%) met the study-defined threshold of <300 pg/mL. This comprised 74 (18.50%) with marginal status and 29 (7.25%) with absolute deficiency; 297 (74.25%) were outside the combined category (**Table 5**).

**Table 5:** Prevalence of vitamin B12 deficiency among study subjects.

Vitamin B12 status	n	%
Combined category (<300 pg/mL)	103	25.75
Marginal status (200 to <300 pg/mL)	74	18.50
Absolute deficiency (<200 pg/mL)	29	7.25
Outside combined category (≥300 pg/mL)	297	74.25

In the count-based univariable analysis, male sex (OR = 1.67; 95% CI: 1.06-2.63), metformin use >5 years (OR = 1.81; 95% CI: 1.07-3.08), and alcohol consumption (OR = 2.03; 95% CI: 1.21-3.40) were associated with the combined outcome. The originally reported anemia estimate was OR = 2.5 (95% CI: 1.3-5.1). Macrocytosis had a highly imprecise reported estimate (OR = 8.9; 95% CI: 1.0-86.3; p = 0.055), which did not meet the p < 0.05 threshold (**Table 3**).

In the originally reported five-variable model, metformin use >5 years (AOR = 1.84; 95% CI: 1.07-3.18) and anemia (AOR = 3.43; 95% CI: 1.62-7.23) were associated with the study-defined outcome. The intervals for male sex, alcohol consumption, and macrocytosis included 1. The macrocytosis interval was particularly wide (0.79-79.37), and its point estimate should not be interpreted as a precise measure of association. **Table 4** retains the reported coefficients; these are not results of a newly fitted or sensitivity-validated model.

## 4 Discussion

In this hospital-based sample, 103 of 400 participants (25.75%) met the study-defined serum vitamin B12 threshold of <300 pg/mL. Most were in the marginal category (74/400, 18.50%), whereas 29 (7.25%) had concentrations <200 pg/mL. The combined proportion should not be interpreted as the prevalence of confirmed functional deficiency or symptomatic disease. Metformin use >5 years and anemia were associated with the outcome in the originally reported adjusted model, but these findings remain exploratory. Male sex and alcohol consumption showed higher proportions in unadjusted comparisons, without a statistically significant adjusted association in the reported model.

The combined proportion was numerically higher than the 18.6% reported by Huynh et al. and the 22.1% reported by Phan et al. in Vietnam, and broadly comparable with the 22.2% reported by Kim et al. in Korea (7, 9, 10). These comparisons are descriptive rather than formal between-study tests. Gundu and Chintha reported 26.36% deficient and a separate 17.83% moderately deficient category (18); their headline proportion should therefore not be equated directly with our combined outcome. Eligibility criteria, biochemical definitions, supplementation, and treatment exposure should be considered before attributing differences to geography or clinical practice.

The study by Vu et al. provides a further example of the importance of supplementation: 79.7% of their elderly metformin-treated participants reported vitamin B12 supplementation (11), whereas current supplement users were excluded from our study. These populations are not directly comparable. Dietary intake was not measured in our study, and we therefore cannot attribute the observed proportion to Vietnamese dietary patterns, differences between northern and southern Vietnam, or alcohol-related nutritional depletion. Likewise, without methylmalonic acid or homocysteine, marginal serum values cannot be assumed to demonstrate functional deficiency in every participant.

The association with metformin use for more than 5 years is compatible with the long-term exposure signal in the randomized trial by de Jager et al. and the longitudinal DPPOS analysis (2, 3). Impaired calcium-dependent absorption offers biological plausibility (4), but these mechanisms were not measured in our study. The observed difference by diabetes duration may also overlap with treatment history rather than represent a distinct causal effect of diabetes itself. Baseline vitamin B12 values were unavailable and every participant received metformin; consequently, neither the temporal sequence of depletion nor the proportion attributable specifically to metformin can be established.

We did not detect significant differences across current daily-dose groups. This contrasts with dose-related associations reported by Kim et al., Gao et al., Huynh et al., and Phan et al. (7–10). Huynh et al. identified dose, rather than treatment duration, as the factor retaining an association in their adjusted models (9). Our findings do not establish that duration is more informative than dose: significance for one exposure and nonsignificance for another are not a test of the difference between their effects. Moreover, daily dose was not included in our reported adjusted model. The dose groups were unequal, with 273 of 400 participants receiving >1500 mg/day, and a current prescription may not reflect cumulative exposure or previous dose changes.

The proportion meeting the combined threshold was higher in men than in women (30.8% versus 21.0%) and in participants reporting alcohol consumption than in those not reporting consumption (37.3% versus 22.7%). Neither variable reached statistical significance in the originally reported adjusted model. These results do not establish an independent determinant, but neither do they prove the absence of an association. Attenuation after adjustment does not establish mediation, explain the sex difference, or demonstrate a more direct causal influence of another covariate.

An alcohol-related contribution is biologically plausible, but evidence from other populations should not be overextended. Gibson et al. found that a controlled alcohol intervention reduced serum vitamin B12 in healthy male volunteers (19). Conversely, Fragasso et al. described alcohol-dependent patients with megaloblastic anemia who responded to vitamin B12 treatment despite normal or borderline serum concentrations (20). Together, these observations suggest that the relationship between alcohol exposure and measured vitamin B12 status is more complex than a uniform decrease in serum concentration. Neither study demonstrates the mechanism underlying the association observed in our patients, whose alcohol exposure was not quantified.

Alcohol consumption in our analysis was represented by a self-reported yes/no variable. It does not distinguish occasional intake from sustained heavy consumption or characterize amount, duration, and drinking pattern. The reported data also do not establish that alcohol use was more frequent among men. We therefore cannot determine whether alcohol explains the higher unadjusted proportion in male participants or whether sex modifies an alcohol-related association. No mediation or sex-by-alcohol interaction result is presented. Shared nutritional, clinical, or treatment-related characteristics and exposure misclassification remain possible explanations, rather than demonstrated mechanisms. Future studies should measure drinking exposure more precisely and evaluate sex-specific associations using adequately powered, prespecified analyses.

Anemia was associated with the outcome in the reported adjusted model (AOR = 3.43; 95% CI: 1.62-7.23), but it is a concurrent clinical correlate rather than an established antecedent risk factor. Cross-sectional measurements cannot determine whether vitamin B12 deficiency contributed to anemia or whether other conditions influenced both. Macrocytosis was not statistically significant in either the univariable (p = 0.055) or reported multivariable analysis (p = 0.078). Its adjusted confidence interval (0.79-79.37) spans approximately two orders of magnitude, precluding a reliable interpretation of the point estimate. Reporting the coefficient for completeness does not establish a robust association or demonstrate that the estimates for the other covariates are insensitive to its inclusion.

Absence of anemia or macrocytosis should not be used to exclude clinically important vitamin B12 deficiency. Lindenbaum et al. documented neuropsychiatric manifestations in patients without these hematological findings (21). In diabetes, neuropathy and biochemical deficiency are not consistently associated in observational studies (5), and the supplementation trial by Didangelos et al. involved a selected symptomatic population (6). These findings provide a clinical rationale for appropriate assessment but do not demonstrate neurological benefit from screening or supplementation in our own cohort, in which such outcomes were not evaluated.

In practice, periodic vitamin B12 assessment should be considered during prolonged metformin treatment, especially when other risk factors are present (12, 13). The >5-year category used here can serve as a pragmatic prompt to prioritize assessment, not a validated threshold or a reason to defer testing earlier. Patients with unexplained anemia or new neuropathic symptoms should be assessed when deficiency is suspected, regardless of treatment duration. This study cannot specify an optimal testing interval or demonstrate a benefit of routine supplementation. A low B12 result does not itself require discontinuation of metformin when the drug remains tolerated and otherwise appropriate (13).

### 4.1 Strengths and limitations

The study includes 400 participants from two hospitals in northern Vietnam and reports absolute and marginal categories separately. These features provide a broader description of serum vitamin B12 status than reporting a single combined percentage. The sample exceeded the calculated minimum for estimating a proportion, but the sample-size calculation did not establish adequate power for every adjusted association or interaction. Increasing sample size also does not remove the selection bias inherent in convenience sampling.

The cross-sectional design precludes temporal or causal conclusions, and hospital-based convenience sampling limits generalizability. All participants received metformin, so excess risk relative to nonuse could not be estimated. Baseline vitamin B12 values, dietary intake, methylmalonic acid, and homocysteine were unavailable. The combined serum threshold includes marginal values and is not equivalent to confirmed functional deficiency. Binary alcohol measurement limits dose-response and sex-specific interpretation; mediation and interaction analyses were not presented. The reported adjusted model omitted diabetes duration and daily metformin dose, leaving potential residual confounding by treatment history. Macrocytosis was estimated with substantial uncertainty. The available results do not establish model stability, including stability after excluding macrocytosis; no such sensitivity result is claimed. The sample-size calculation addressed estimation of a proportion rather than the precision of every adjusted coefficient. Longitudinal research with detailed exposure measurement, functional biomarkers, and documented analytical plans is needed.

## 5 Conclusions

Low serum vitamin B12, including marginal values, was present in 103 of 400 participants (25.75%). Metformin use >5 years and anemia were associated with the combined outcome in the originally reported exploratory adjusted model. These results should be interpreted as clinical associations, not established causal risk factors or a validated prediction rule.

Periodic vitamin B12 assessment merits consideration during long-term metformin therapy. Use >5 years can help prioritize review of vitamin B12 status, while suggestive anemia or neuropathic symptoms warrant earlier assessment when deficiency is suspected. The study does not validate a five-year screening threshold, establish an optimal testing interval, show that duration is a better predictor than dose, or demonstrate stability of the adjusted estimates under alternative model specifications.

## Data Availability

The raw data supporting the conclusions of this article will be made available by the authors, without undue reservation.
